# Changes in survival probabilities and mortality risks among population living with Down syndrome born 1967–2018: a Norwegian registry-based study

**DOI:** 10.1186/s12889-025-25326-z

**Published:** 2025-12-22

**Authors:** Teferi Mekonnen, Dana Kristjansson, Bernt Bratsberg, Hans-Peter Kohler, Geir Selbæk, Frode Kibsgaard Larsen, Ellen Melbye Langballe, Jeanette Engeland, Øyvind Kirkevold, Asta Kristine Håberg, Bjørn Heine Strand, Vegard Skirbekk

**Affiliations:** 1 Department for Physical Health and Aging, Norwegian Institute of Public Health, Oslo, Norway; 2 Norwegian National Centre for Ageing and Health, Vestfold Hospital Trust, Tønsberg, Norway; 3 Centre for Fertility and Health, Norwegian Institute of Public Health, Oslo, Norway; 4 Ragnar Frisch Center for Economic Research, Oslo, Norway; 5 Department of Geriatric Medicine, Oslo University Hospital, Oslo, Norway; 6 Faculty of Medicine, University of Oslo, Oslo, Norway; 7 Population Aging Research Center and Department of Sociology, University of Pennsylvania, Philadelphia, PA USA; 8 Department of Genetics and Bioinformatics, Norwegian Institute of Public Health, Oslo, Norway; 9 Department of Neuromedicine and Movement Science, Faculty of Medicine and Health Sciences, Norwegian University of Science and Technology, Trondheim, Norway; 10 Center for Innovation, Medical Equipment, and Technology, MiDT National Research Center, St. Olav University Hospital, Trondheim University Hospital, Trondheim, Norway; 11 Department of Health Sciences, Faculty of Medicine and Health Sciences, Norwegian University of Science and Technology, Trondheim, Norway; 12 Department of Psychology, University of Oslo, Oslo, Norway

**Keywords:** Down syndrome, Survival, Mortality, Cohort differences

## Abstract

**Background:**

While medical advancements and increased awareness have improved the life expectancy of individuals with Down syndrome (DS), studies on cohort-specific differences in mortality risk and long-term survival outcomes among individuals with DS remain limited. We assessed cohort differences in survival probabilities and mortality risk among individuals with DS born 1967–2018, using Norwegian national registry data.

**Methods:**

Utilizing the Medical Birth Registry of Norway linked with additional registries, we included 3,014,455 eligible individuals. Kaplan–Meier survival curves and mortality rates were employed to examine birth cohort differences in survival probability between individuals with DS and the rest of the population up to age 40 years. A flexible parametric survival model was used to identify mortality risk factors among individuals with DS.

**Results:**

Among 3,014,455 individuals, 3,484 (1.2 per 1000) were diagnosed with DS. Across birth cohorts, the most significant survival gap occurred in early childhood (less than 5 years) when compared to individuals without DS. Mortality rates for individuals with DS were consistently higher than the rest of the population across all birth cohorts. In analyses of follow-up birth to age 40 years, individuals born from 1967 to 1976 demonstrated the highest mortality risk (HR: 6.35) followed by those born from 1977 to 1986 (HR: 3.83) and from 1987 to 1996 (HR: 2.31) compared to those born between 2007 and 2018. Low birth weight (< 2,500 g) was associated with increased mortality risk (HR: 1.47) compared to those born with birth weight of 2500 g or more. Individuals born into households with income below the median showed a higher mortality risk (HR: 1.41) compared to those from higher-income households. Having one or more siblings also correlated with increased mortality risk (HR: 1.46) compared to having none. In sub-analyses restricted to follow-up from birth to five years, birth year remained a significant predictor of mortality risk for individuals with Down syndrome (DS). Compared with those born between 2007 and 2018, highest mortality risk was observed for births from 1967 to 1976 (HR: 6.18), 1977 to 1986 (HR: 3.61), and 1987 to 1996 (HR: 2.06). Low birth weight (HR: 1.66) and having one or more siblings (HR:1.56) was also showed a greater mortality risk. For the follow-up period from ages 6 to 40 years, those born into lower-income households experienced increased mortality risk (HR: 2.16).

**Conclusions:**

This study found that individuals with DS experience significantly lower survival, particularly (with the largest gap) in early childhood. Being born in earlier birth cohort, low birth weight, lower household income, and having siblings were associated with higher mortality risk among individuals with DS. These findings underscore the need for targeted interventions (and support) to improve survival outcomes for individuals with DS.

**Supplementary Information:**

The online version contains supplementary material available at 10.1186/s12889-025-25326-z.

## Introduction

Down syndrome (DS) is a genetic disorder caused by the presence of an extra full or partial copy of chromosome 21. Live births of children with DS occur in approximately 1 in 700 in the United States and 1 in 1,000 in Europe; DS is the most common congenital cause of intellectual disability [1–4]. DS is also associated with multiple complex comorbidities, particularly heart defects, diabetes, leukemia, and a higher risk of Alzheimer’s disease [5].

While advances in medical care and early intervention have improved the overall survival for individuals with DS [6], there remains a need to understand the relationship of different sociodemographic factors with DS survival rates using population level data. Social determinants of health, such as familial support, education, and income level, have been found to influence several DS-associated comorbidities, including leukemia [7] and Alzheimer’s disease [8, 9]. However, there is scant research focused on the relationship between social factors and DS survival rates.

Nine out of ten children with DS live with their families, and individuals with DS rely on their support throughout childhood and into adulthood [10, 11]. Families play a central role in advocating for personalized care for children and adults with DS [12]. Additionally, socioeconomic status, family structure, and access to healthcare can play a pivotal role in DS survival. Children with DS from low-income families were nearly three times more likely to have unmet medical needs and twice as likely to have unmet needs for family support services [10].

Families with a child with DS also reported positive aspects, with closer sibling relationships, more frequent social contact, and less depressive symptoms [13]. These parents often find meaningful pathways forward, reporting high levels of love, pride, and positive outlooks on life [14]. For the individual with DS in particular, the support of one’s family can significantly enhance well-being, offering emotional assistance, aid in daily activities, and access to essential medical services. Close kin, such as family members, contribute to a sense of purpose and encourage health-promoting behaviors, leading to improved health outcomes [15–18]. Positive family dynamics influence lifestyle, preventive behaviors, and care provision, and impact life expectancy [19, 20]. This care is particularly important for those with DS, who may rely on familial support to ensure a healthier lifestyle, tackle health issues, and navigate a complex health system.

This study investigates cohort differences in survival probabilities and mortality risks among individuals with Down syndrome in Norway, born between 1967 and 2018, and compares them with those of the rest of the population, using data from the Norwegian Medical Birth Registry and other associated Norwegian registries. We also examined the role of sociodemographic including number of siblings and early life factors in mortality risk among individuals with Down syndrome.

## Methods

### Study design, setting and participants

A prospective population based observational cohort design using data from the Norwegian Medical Birth Registries (MBRN), Norwegian Cause of Death Registry, Statistics Norway (i.e., population register) and the events database for welfare benefits (FD-Trygd) was used. Norwegian national registries achieve nearly 100% completeness and accuracy in vital statistics, offering a reliable resource for epidemiological studies. Information about sociodemographic characteristics, survival status was obtained from the population registry of Norway and the Norwegian cause of death registry, respectively. A total of 3,014,455 eligible participants, including both those with DS (*n* = 3,484) and without DS (*n* = 3,010,971), were included in our study.

### Definition of study variables

#### Down syndrome status

Information about DS status was obtained from the MBRN and FD-trygd databases and defined as “Yes” if diagnosed with DS vs. “No” if they did not. Additional cases of DS were included from the events database for welfare benefits, which were based on ICD9 and ICD10.

#### Outcome variable and follow up periods

Study participants' survival status (i.e., dead vs. alive) was determined using information from the Cause of Death Registry reported in months of birth and death. For the main analysis, we followed the study population from birth to age 40 years. Due to observed differences in mortality between younger and older born participants, a secondary analysis was conducted by restricting the follow-up period to ages 0–5 years and 6–40 years. Analyzing survival separately for individuals up to age 5 years and beyond 5 years separately will allow us to separate early-life congenital and medical challenges from socio-developmental influences on survival among those born with DS. Additionally, survival probability estimates up to age 40 for individuals with and without DS offer insights into distinct health challenges, including transitions from early childhood to adolescence, early adulthood, mid-adulthood.

### Birth cohort and other sociodemographic, perinatal and early postnatal characteristics

Information on participants' birth year was obtained from the population registry and was defined based on the follow-up periods. For the follow up birth to 40 years, birth to 5 years, and 6 years to 40 years birth cohort was defined as born 1967–1976, 1977–1986, 1987–1996, 1997–2006, 2007–2018. For the follow-up period of 6–40 years, we defined birth cohorts as those born in 1967–1976, 1977–1986, 1987–1996, and 1997–2018. The cohorts for 1997–2006 and 2007–2018 were combined into a single group because the number of deaths in the 2007–2018 cohort was very low.

Gestational age was defined as born ≥ 38 vs. < 38 weeks) obtained from the MBRN. Birth weight was dichotomized to ≥ 2,500 g vs. < 2,500 g.

Information on whether they have siblings or not were defined using information from population registry (Statistics Norway). Sibling status was defined as “Yes” if they have one or more sibling from the same parents’ and “No” if they had no siblings.

Parental characteristics were assessed using information on whether their mother and father is alive, or dead using information from the population registers and causes of death registry. Death of mother was defined as “No” if mother was alive at one point between 1967–2021 vs. “Yes” if mother died during the follow up in the same period. Similarly, individuals experiencing death of father was defined as “No” if their father was alive during 1967–2021 vs. “Yes” if their father died during the follow up period. Mother’s age at birth was obtained from the medical birth registry and was recorded as < 30 years vs. ≥ 30 years.

Mother’s marital status at birth was defined as “living with a partner” if married/cohabitant/registered partner vs. “not living with a partner” if “unmarried/single/divorced/widowed and separated”.

Parental household income at time of birth, inflated to 2018 NOK using the consumer price index, was determined using data from Statistics Norway, classified based on the median income of the participants' parents, which was 503,495 NOK were categorized as having an income “below the median” if the combined parental income was < 503,495 NOK, and as having a “median or above” income if ≥ 503,495 NOK.

### Statistical analysis

Survival probability curves for the whole population and by DS status were used to describe survival characteristics of individuals living with and without DS. Survival probabilities by birth cohort were explored using Kaplan–Meier curves. Cohort trends in mortality rates per 1,000 person-years by birth year were investigated. A flexible parametric model was fitted to identify factors associated with mortality among individuals living with DS. For the main analysis, survival probabilities for follow-up from birth to 40 years were computed. In addition, given the highest mortality rates and survival gaps during early and later life, a secondary analysis was performed by restricting the follow-up period to birth to 5 years and 6–40 years. To identify factors associated with mortality among population living with DS across each follow-up period, we conducted analyses using two models: (1) a confounder-adjusted model, accounting for sex and birth year, and (2) a fully adjusted model, encompassing all available risk factors and confounders. These models were used to assess the attenuation of birth year estimates by potential mediators. Adjusted hazard ratios (HRs) with 95% confidence intervals (CIs) were reported. A *p*-value of less than 0.05 was considered statistically significant. Given the presence of missing information for some covariates, we assessed model fit by comparing the AIC for Model 1, which had no missing data, with a complete case analysis (Model 2).

## Results

### Baseline characteristics of the study population

Of 3,014,455 eligible participants, 3,484 (0.12%) were diagnosed with Down syndrome (DS). Table 1 compares characteristics between individuals with and without DS. The proportion of individuals with Down syndrome increased slightly in more recent birth cohorts, rising from 17% in 1967–1976 to 25% in 2007–2018. A higher proportion of males (53% vs. 51%), births to married women (93% vs. 91%), births to women aged 30 years and above (67% vs. 38%), and families with median or below-median income levels (46% vs. 50%) were observed among individuals with DS. Higher proportion experienced the death of their mother (8.5% vs. 5.4%) or father (15% vs. 11%). Additionally, 67% were born ≥ 38 weeks of gestation compared to 90% without DS. There were 83% of individuals who had DS who had a birth weight ≥ 2,500 g compared to 95% of individuals without DS (Table 1).

**Table 1:**
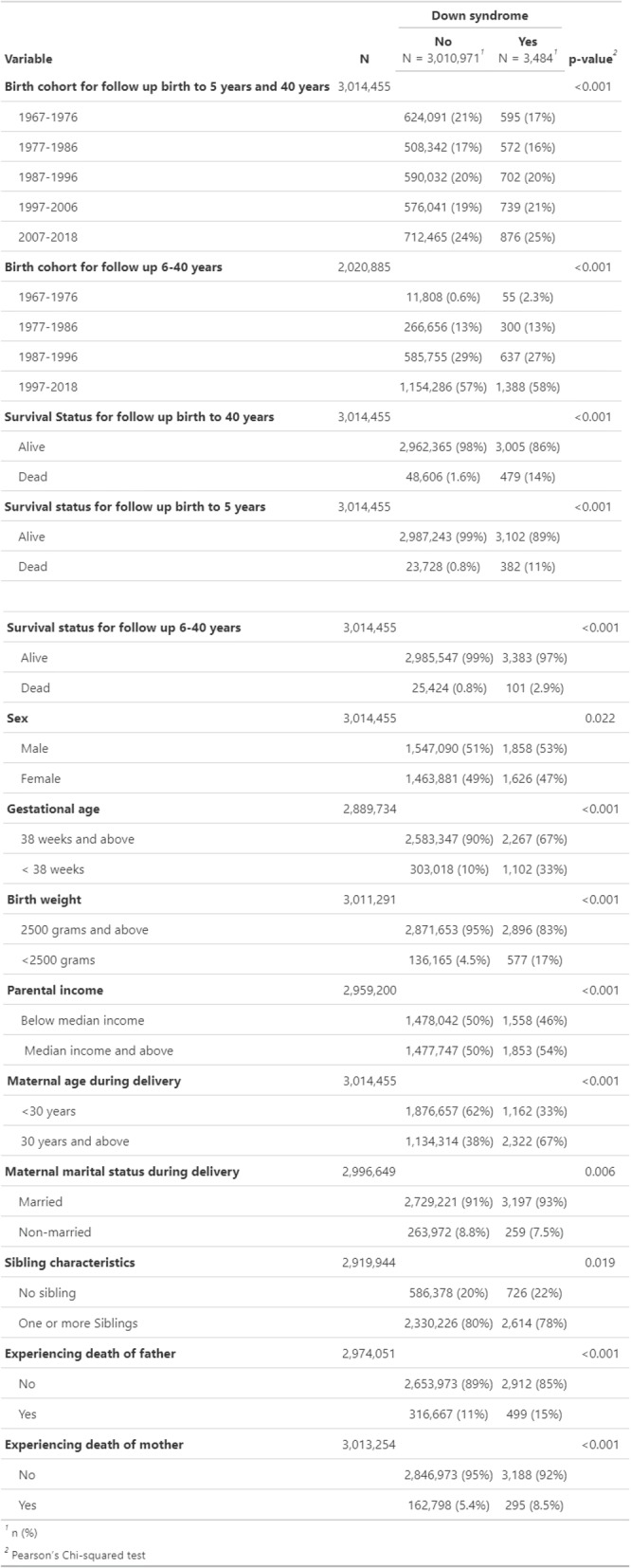
Characteristics of study participants

Gestational age, birth weight, parental income, maternal age at delivery, maternal marital status at delivery, sibling characteristics and experiencing death (of the mother or father) contain missing values, and the sample size varies across variables

### Survival characteristics among individuals living with and without Down syndrome

**Fig. 1:**
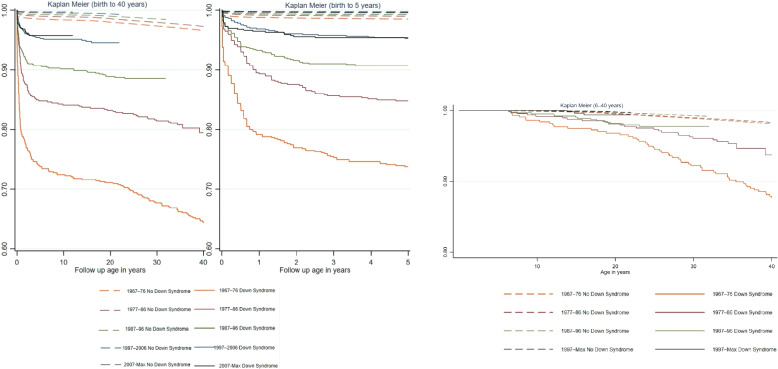
Survival probability by birth cohort and Down syndrome status among participants for follow up birth to 40 years, birth to 5 years and 6–40 years

This figure presents Kaplan–Meier survival curves for individuals with and without DS, stratified by birth cohort (Fig. 1). The survival probability is consistently lower for individuals with DS across follow up periods oof birth to 40 years, birth to 5 years and 6–40 years with the most pronounced difference observed in early childhood, where the survival gap is largest. Our results showed statistically significant survival differences among DS across cohorts for both follow up periods (*p* < 0.001).

**Fig. 2:**
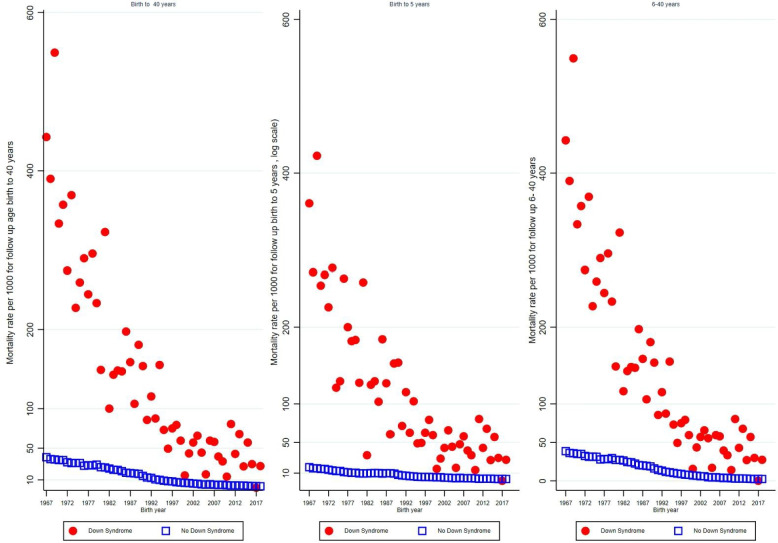
Trends in mortality rate per 1000 by birth cohort and Down syndrome status among participants with follow up age birth to 40 years, birth to 5 years and 6 to 40 years

Figure 2 illustrates the trends in mortality rates per 1,000 by birth cohort and DS status for the follow-up periods: from birth to 40 years, birth to 5 years and 6–40 years, respectively (Fig. 2). Among individuals with DS, the mortality rate before age 40 remained persistently high across the 1967–2018 birth cohorts, ranging approximately between 14 and 549 per 1,000 with the highest mortality occurred until early 1990th. Conversely, mortality in the same age range among individuals without DS remained low and relatively stable at about 2–39 per 1,000. The mortality rate before age 5 among individuals with DS showed a marked decline across birth cohorts, ranging approximately between 14 per 1,000 to 423 per 1,000 live births during the follow up. In contrast, mortality among individuals without DS also declined steadily over time but remained substantially lower throughout, ranging from 2 per 1,000 to 18 per 1,000. For the follow-up period of 6–40 years, among individuals with DS, remained persistently high across the 1967–2018 birth cohorts, ranging approximately between 14 and 549 per 1,000. A higher mortality was observed until the 1990s. Conversely, in the same age range, mortality among individuals without DS remained low and relatively stable, at around 2–39 per 1,000.

### Associated with mortality among populations with Downs syndrome

The table illustrates factors associated with mortality in individuals with Down syndrome for the follow up restricted to the age of 40 years, birth to 5 years and 6–40 years (Table 2). Our results showed that birth year is the strongest risk factors for mortality in this population. Compared to those born between 2007–2018, individuals born from 1967 to 1976 have the highest hazard ratio (HR: 6.35 [3.90–10.35]), followed by those born from 1977 to 1986 (HR: 3.83 [2.41–6.08]) and from 1987 to 1996 (HR: 2.31 [1.44–3.69]). A birth weight of less than 2,500 g is associated with increased mortality risk (HR: 1.47 [1.13–1.93]) compared to those with a birth weight of 2,500 g or more. Individuals born into households with income below the median had a higher mortality risk (HR: 1.41 [1.11–1.79]), compared to those whose parents earned above median income. Having one or more siblings was linked to higher mortality (HR: 1.46 [1.13–1.89]), compared to those with no siblings. Other factors such as sex, gestational age under 38 weeks, maternal age of 30 years or older at pregnancy, unmarried maternal status, and the death of the mother and father during the follow-up period were not statistically significant (Table 2).

**Table 2:**
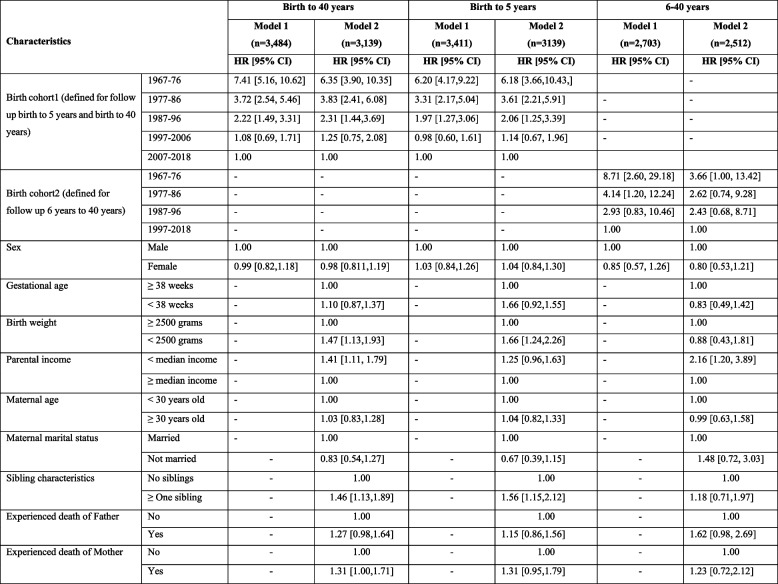
Factors associated with mortality among individuals with Down syndrome for follow up birth to 40 years, birth to 5 years and 6–40 years, based on a confounder-adjusted model (Model 1) and a fully adjusted model (Model 2)

In a follow-up restricted to birth to five years, birth year remains the most significant mortality predictor among those with DS. Compared to those born between 2007–2018, the hazard ratios are higher for those born 1967–1976 (HR: 6.18 [3.66–10.43]), born 1977–1986 (HR: 3.61 [2.21–5.91]), and born 1987–1996 (HR: 2.06 [1.25–3.39]). Birth weights under 2,500 g still present a greater mortality risk (HR: 1.66 [1.24–2.26]) than those with a birth weight of 2,500 g or more. Having one or more siblings was linked to higher mortality (HR: 1.56 [1.15–2.12]), compared to those with no siblings (Table 2).

For the follow up 6–40 years, those born into households with income below the median had a higher mortality risk associated with higher mortality risk (HR: 2.16 [1.20–3.89]) (Table 2).

Our regression-based models indicated that the increased mortality risk observed among individuals with DS born in the earlier cohorts (1967–1976) for follow-up period from birth to 5 years could be partly explained by covariates such as gestational age, birth weight, household income, maternal age at delivery, maternal marital status, sibling characteristics, and parental death (Table 2). In addition, the observed increase in hazard ratios for the intermediate cohorts (1977–1986 and 1987–1996) compared to 1997–2006 in our fully adjusted model may reflect substantial medical advancements including perinatal and postnatal screening and pediatric care that likely altered health outcomes.

## Discussion

This study revealed cohort differences in survival among individuals with DS born between 1967 and 2021 with an improved survival among later born cohorts. Across all cohorts, individuals with DS consistently exhibited lower survival probabilities compared to those without the condition, with the survival gap being most pronounced during early childhood. While mortality rates among individuals with DS declined significantly in early life (i.e., before age 5) across birth cohorts, they remained relatively high before age 40. In contrast, individuals without DS experienced better survival and lower mortality rates over the same time span. Low birth weight, having parents with low income, and having one or more siblings were identified as factors that increase mortality among individuals with DS.

The observed improvement in childhood survival rates among more recent birth cohorts with DS is an encouraging trend, reflecting advancements in medical care, early interventions, and improved social support for individuals with the condition. These findings are consistent with trends observed in other high-income countries, including Denmark [21], Sweden [22], the United Kingdom [23], and the United States [24], where access to specialized healthcare and medical innovations has expanded in recent decades. However, survival among individuals with DS remains lower than that of the whole population, indicating that despite overall progress, significant disparities persist. The continued mortality gap highlights the need for sustained efforts to identify and address the health challenges faced by populations living with DS.

Our analyses found a significant association between parental income and DS survival probability, with those born to parents earning below the median income exhibiting a higher risk of mortality. This finding aligns with a growing body of evidence underscoring the profound impact of socioeconomic factors on health outcomes in populations with developmental disabilities [25, 26]. Socioeconomic disadvantages can limit access to quality healthcare services, early interventions, and supportive resources, thereby exacerbating health disparities and mortality risk.

Our findings indicate that individuals with DS born with a low birth weight (< 2,500 g) face a significantly increased risk of mortality than their peers born with normal birth weight. The observed association aligns with previous research demonstrating that low birth weight is a critical determinant of mortality among people with DS [6, 27, 28], often reflecting underlying fetal and neonatal complications and increased vulnerability during infancy and childhood. Furthermore, low birth weight and household income are often interrelated, as socioeconomic deprivation is associated with increased rates of low birth weight [29, 30], which, in turn, contributes to adverse health outcomes. These findings underscore the need for comprehensive approaches addressing both biological and social determinants of health to improve long-term survival among individuals with DS.

The significant decline in survival observed among those with DS during early in life in our study may be attributed to the compounded health challenges faced by infants with DS, including congenital heart defects, respiratory issues, and digestive problems associated with the syndrome [31–33]. Furthermore, secondary analysis indicated that the elevated risk of earlier born individuals partly attenuated after accounting for parental income, maternal age, and birth weight. These findings highlight socioeconomic disadvantages and perinatal vulnerabilities, such as low parental income and low birth weight, play important roles in DS survival disparities.

Our findings indicated that individuals with DS who have one or more siblings demonstrated higher mortality compared to those with no siblings. This may be partly explained by the fact that only children may receive more focused parental attention and resources, such as more joint time, more supervision and help with preventive behaviour, use of healthcare services, financial support, and emotional investment, compared to children with siblings, For individuals with DS, who often require additional medical, educational, and therapeutic support, concentrated parental resources could theoretically enhance access to care, leading to better health management. However, no studies explicitly compare the health outcomes of individuals with DS with and without siblings. Although siblings can positively influence social and emotional development in individuals with DS, it doesn’t necessarily compensate for the parental time spent on helping them with everyday issues, helping them with physical health or attention dilution. Conversely, siblings might compete for parental resources, potentially reducing the time available for specialized care, though this effect is speculative.

While this study provides important insights, some limitations should be noted. First, the data are derived from registry-based sources, which, while comprehensive, may not capture all relevant sociodemographic or health-related factors. Finally, while the study explores the influence of socioeconomic and familial factors in Norway, the familial structure, social support, and healthcare access that influences survival and mortality differ in many countries, particularly low- to middle-income countries which may have a stronger reliance on familial caretaking roles with less healthcare accessibility. In addition, the parents’ life course marital status is not documented. While some studies suggest higher divorce rates among parents of children who have disabilities compared to those whose children without have disabilities [34], other studies have found the opposite [35]. In addition, the results from our flexible parametric model are based on a complete case analysis. Although the proportion of missing data for the variables included in the model was low and sensitivity analyses indicated that the exclusion of incomplete cases did not materially affect the results, the possibility of residual bias due to missing data cannot be entirely ruled out.

In conclusion, there have been significant improvements in survival among individuals with DS from 1967 to 2021. Disparities based on socioeconomic status and perinatal risk factors affect the likelihood of early death among individuals with DS and highlight the need for continued efforts to address these inequalities. The findings underscore the importance of economic support to improve survival with DS. Targeted interventions are required to tackle socioeconomic disparities, and future research should focus on strategies to support individuals with DS and their families in overcoming these challenges.

## Supplementary Information

Supplementary Material 1.

## Data Availability

The data used in the current study are available after approval by the Regional Committee for Medical and Health Research Ethics. The data is not publicly available but can be granted upon application to the respective data owners and requires approval from REK.
