# Supplementary material for: Changes in survival probabilities and mortality risks among population living with Down syndrome born 1967–2018: a Norwegian registry-based study
Source: BMC Public Health. 2025 Dec 22;25:4272. doi: 10.1186/s12889-025-25326-z (PMC12723947; doi:10.1186/s12889-025-25326-z)
Supplement: Supplementary file 1 — Supplementary Material 1. [file 12889_2025_25326_MOESM1_ESM.docx]

**Changes in survival probabilities and mortality risks among population living with Down syndrome born 1967-2018: a Norwegian registry-based study**

*Teferi Mekonnen^1^, *Dana Kristjansson^3,8^, Bernt Bratsberg^3,4^, Hans-Peter Kohler^7^, Geir Selbæk^2,5,6^, Frode Kibsgaard Larsen^2,10^, Ellen Melbye Langballe^2^, Jeanette Engeland^2^, Øyvind Kirkevold^11^, Asta Kristine Håberg^9,10^ Bjørn Heine Strand^1,2,5^, Vegard Skirbekk^1,2,3,12^

Affiliations

^1^Department for Physical Health and Aging, Norwegian Institute of Public Health, Oslo, Norway

^2^Norwegian National Centre for Ageing and Health, Vestfold Hospital Trust, Tønsberg, Norway

^3^Centre for Fertility and Health, Norwegian Institute of Public Health, Oslo, Norway

^4^Ragnar Frisch Center for Economic Research, Oslo, Norway

^5^Department of Geriatric Medicine, Oslo University Hospital, Oslo, Norway

^6^Faculty of Medicine, University of Oslo, Oslo, Norway

^7^Population Aging Research Center and Department of Sociology, University of Pennsylvania, Philadelphia, PA, USA

^8^Department of Genetics and Bioinformatics, Norwegian Institute of Public Health, Oslo, Norway

^9^Department of Neuromedicine and Movement Science, Faculty of Medicine and Health Sciences, Norwegian University of Science and Technology, Trondheim, Norway

^10^MiDT national research center, Center for innovation, medical equipment, and technology, St. Olav University Hospital, Trondheim University Hospital, Trondheim, Norway

^11^Department of Health Sciences, Faculty of Medicine and Health Sciences, Norwegian University of Science and Technology, Trondheim, Norway

^12^Department of Psychology, University of Oslo, Oslo, Norway

*Shared first authorship

Corresponding author: Teferi Mekonnen (PhD)

Email: [teferimekonnen.yitayew@fhi.no](mailto:teferimekonnen.yitayew@fhi.no)

**Abstract**
**Background:** While medical advancements and increased awareness have improved the life expectancy of individuals with Down syndrome (DS), studies on cohort-specific differences in mortality risk and long-term survival outcomes among individuals with DS remain limited. We assessed cohort differences in survival probabilities and mortality risk among individuals with DS born 1967-2018, using Norwegian national registry data.

**Methods:** Utilizing the Medical Birth Registry of Norway linked with additional registries, we included 3,014,455 eligible individuals. Kaplan-Meier survival curves and mortality rates were employed to examine birth cohort differences in survival probability between individuals with DS and the rest of the population up to age 40 years. A flexible parametric survival model was used to identify mortality risk factors among individuals with DS.
**Results:** Among 3,014,455 individuals, 3,484 (1.2 per 1000) were diagnosed with DS. Across birth cohorts, the most significant survival gap occurred in early childhood (less than 5 years) when compared to individuals without DS. Mortality rates for individuals with DS were consistently higher than the rest of the population across all birth cohorts.  In analyses of follow-up birth to age 40 years, individuals born from 1967 to 1976 demonstrated the highest mortality risk (HR: 6.35) followed by those born from 1977 to 1986 (HR: 3.83) and from 1987 to 1996 (HR: 2.31) compared to those born between 2007 and 2018. Low birth weight (<2,500 grams) was associated with increased mortality risk (HR: 1.47) compared to those born with birth weight of 2500 grams or more. Individuals born into households with income below the median showed a higher mortality risk (HR: 1.41) compared to those from higher-income households. Having one or more siblings also correlated with increased mortality risk (HR: 1.46) compared to having none. In sub-analyses restricted to follow-up from birth to five years, birth year remained a significant predictor of mortality risk for individuals with Down syndrome (DS). Compared with those born between 2007 and 2018, highest mortality risk was observed for births from 1967 to 1976 (HR: 6.18), 1977 to 1986 (HR: 3.61), and 1987 to 1996 (HR: 2.06). Low birth weight (HR: 1.66) and having one or more siblings (HR:1.56) was also showed a greater mortality risk. For the follow-up period from ages 6 to 40 years, those born into lower-income households experienced increased mortality risk (HR: 2.16).

**Conclusions:** This study found that individuals with DS experience significantly lower survival, particularly (with the largest gap) in early childhood. Being born in earlier birth cohort, low birth weight, lower household income, and having siblings were associated with higher mortality risk among individuals with DS. These findings underscore the need for targeted interventions (and support) to improve survival outcomes for individuals with DS.

**Keywords**

Down syndrome; Survival; Mortality; Cohort differences

**Introduction**

Down syndrome (DS) is a genetic disorder caused by the presence of an extra full or partial copy of chromosome 21. Live births of children with DS occur in approximately 1 in 700 in the United States and 1 in 1,000 in Europe; DS is the most common congenital cause of intellectual disability [1-4]. DS is also associated with multiple complex comorbidities, particularly heart defects, diabetes, leukemia, and a higher risk of Alzheimer’s disease [5].

While advances in medical care and early intervention have improved the overall survival for individuals with DS [6], there remains a need to understand the relationship of different sociodemographic factors with DS survival rates using population level data. Social determinants of health, such as familial support, education, and income level, have been found to influence several DS-associated comorbidities, including leukemia [7] and Alzheimer’s disease [8, 9]. However, there is scant research focused on the relationship between social factors and DS survival rates.

Nine out of ten children with DS live with their families, and individuals with DS rely on their support throughout childhood and into adulthood [10, 11]. Families play a central role in advocating for personalized care for children and adults with DS [12]. Additionally, socioeconomic status, family structure, and access to healthcare can play a pivotal role in DS survival. Children with DS from low-income families were nearly three times more likely to have unmet medical needs and twice as likely to have unmet needs for family support services [10].

Families with a child with DS also reported positive aspects, with closer sibling relationships, more frequent social contact, and less depressive symptoms [13]. These parents often find meaningful pathways forward, reporting high levels of love, pride, and positive outlooks on life [14]. For the individual with DS in particular, the support of one’s family can significantly enhance well-being, offering emotional assistance, aid in daily activities, and access to essential medical services. Close kin, such as family members, contribute to a sense of purpose and encourage health-promoting behaviors, leading to improved health outcomes [15-18]. Positive family dynamics influence lifestyle, preventive behaviors, and care provision, and impact life expectancy [19, 20]. This care is particularly important for those with DS, who may rely on familial support to ensure a healthier lifestyle, tackle health issues, and navigate a complex health system.

This study investigates cohort differences in survival probabilities and mortality risks among individuals with Down syndrome in Norway, born between 1967 and 2018, and compares them with those of the rest of the population, using data from the Norwegian Medical Birth Registry and other associated Norwegian registries. We also examined the role of sociodemographic including number of siblings and early life factors in mortality risk among individuals with Down syndrome.

**Methods**

**Study design, setting and participants**

A prospective population based observational cohort design using data from the Norwegian Medical Birth Registries (MBRN), Norwegian Cause of Death Registry, Statistics Norway (i.e., population register) and the events database for welfare benefits (FD-Trygd) was used. Norwegian national registries achieve nearly 100% completeness and accuracy in vital statistics, offering a reliable resource for epidemiological studies. Information about sociodemographic characteristics, survival status was obtained from the population registry of Norway and the Norwegian cause of death registry, respectively. A total of 3,014,455 eligible participants, including both those with DS (n=3,484) and without DS (n=3,010,971), were included in our study.

**Definition of study variables**

*Down syndrome status*

Information about DS status was obtained from the MBRN and FD-trygd databases and defined as “Yes” if diagnosed with DS vs. “No” if they did not. Additional cases of DS were included from the events database for welfare benefits, which were based on ICD9 and ICD10.

*Outcome variable and follow up periods*

Study participants' survival status (i.e., dead vs. alive) was determined using information from the Cause of Death Registry reported in months of birth and death. For the main analysis, we followed the study population from birth to age 40 years. Due to observed differences in mortality between younger and older born participants, a secondary analysis was conducted by restricting the follow-up period to ages 0–5 years and 6-40 years. Analyzing survival separately for individuals up to age 5 years and beyond 5 years separately will allow us to separate early-life congenital and medical challenges from socio-developmental influences on survival among those born with DS. Additionally, survival probability estimates up to age 40 for individuals with and without DS offer insights into distinct health challenges, including transitions from early childhood to adolescence, early adulthood, mid-adulthood.

**Birth cohort and other sociodemographic, perinatal and early postnatal characteristics**

Information on participants' birth year was obtained from the population registry and was defined based on the follow-up periods. For the follow up birth to 40 years, birth to 5 years, and 6 years to 40 years birth cohort was defined as born 1967-1976, 1977-1986, 1987-1996, 1997-2006, 2007-2018. For the follow-up period of 6–40 years, we defined birth cohorts as those born in 1967–1976, 1977–1986, 1987–1996, and 1997–2018. The cohorts for 1997–2006 and 2007–2018 were combined into a single group because the number of deaths in the 2007–2018 cohort was very low

Gestational age was defined as born ≥ 38 vs. < 38 weeks) obtained from the MBRN. Birth weight was dichotomized to ≥ 2,500 grams vs. < 2,500 grams.

Information on whether they have siblings or not were defined using information from population registry (Statistics Norway). Sibling status was defined as “Yes” if they have one or more sibling from the same parents’ and “No” if they had no siblings.

Parental characteristics were assessed using information on whether their mother and father is alive, or dead using information from the population registers and causes of death registry. Death of mother was defined as “No” if mother was alive at one point between 1967-2021 vs. “Yes” if mother died during the follow up in the same period. Similarly, individuals experiencing death of father was defined as “No” if their father was alive during 1967-2021 vs. “Yes” if their father died during the follow up period. Mother’s age at birth was obtained from the medical birth registry and was recorded as < 30 years vs. ≥30 years.

Mother’s marital status at birth was defined as “living with a partner” if married/ cohabitant/ registered partner vs. “not living with a partner” if “unmarried/single/divorced/widowed and separated”.

Parental household income at time of birth, inflated to 2018 NOK using the consumer price index, was determined using data from Statistics Norway, classified based on the median income of the participants' parents, which was 503,495 NOK were categorized as having an income “below the median” if the combined parental income was < 503,495 NOK, and as having a “median or above” income if ≥ 503,495 NOK.

**Statistical analysis**

Survival probability curves for the whole population and by DS status were used to describe survival characteristics of individuals living with and without DS. Survival probabilities by birth cohort were explored using Kaplan-Meier curves. Cohort trends in mortality rates per 1,000 person-years by birth year were investigated. A flexible parametric model was fitted to identify factors associated with mortality among individuals living with DS. For the main analysis, survival probabilities for follow-up from birth to 40 years were computed. In addition, given the highest mortality rates and survival gaps during early and later life, a secondary analysis was performed by restricting the follow-up period to birth to 5 years and 6–40 years. To identify factors associated with mortality among population living with DS across each follow-up period, we conducted analyses using two models: (1) a confounder-adjusted model, accounting for sex and birth year, and (2) a fully adjusted model, encompassing all available risk factors and confounders. These models were used to assess the attenuation of birth year estimates by potential mediators. Adjusted hazard ratios (HRs) with 95% confidence intervals (CIs) were reported. A p-value of less than 0.05 was considered statistically significant. Given the presence of missing information for some covariates, we assessed model fit by comparing the AIC for Model 1, which had no missing data, with a complete case analysis(Model 2). .

**Results**

**Baseline characteristics of the study population**

Of 3,014,455 eligible participants, 3,484 (0.12%) were diagnosed with Down syndrome (DS). Table 1 compares characteristics between individuals with and without DS. The proportion of individuals with Down syndrome increased slightly in more recent birth cohorts, rising from 17% in 1967–1976 to 25% in 2007–2018. A higher proportion of males (53% vs. 51%), births to married women (93% vs. 91%), births to women aged 30 years and above (67% vs. 38%), and families with median or below-median income levels (46% vs. 50%) were observed among individuals with DS. Higher proportion experienced the death of their mother (8.5% vs. 5.4%) or father (15% vs. 11%). Additionally, 67% were born ≥ 38 weeks of gestation compared to 90% without DS. There were 83% of individuals who had DS who had a birth weight ≥ 2,500 g compared to 95% of individuals without DS. (Table 1).

Table 1: characteristics of study participants

**Survival characteristics among individuals living with and without Down syndrome.**

This figure presents Kaplan-Meier survival curves for individuals with and without DS, stratified by birth cohort (Figure 1). The survival probability is consistently lower for individuals with DS across follow up periods oof birth to 40 years, birth to 5 years and 6-40 years with the most pronounced difference observed in early childhood, where the survival gap is largest. Our results showed statistically significant survival differences among DS across cohorts for both follow up periods (p < 0.001).

Figure 1: Survival probability by birth cohort and Down syndrome status among participants for follow up birth to 40 years, birth to 5 years and 6-40 years.

Figure 2 illustrates the trends in mortality rates per 1,000 by birth cohort and DS status for the follow-up periods: from birth to 40 years, birth to 5 years and 6-40 years, respectively (Figure 2). Among individuals with DS, the mortality rate before age 40 remained persistently high across the 1967–2018 birth cohorts, ranging approximately between 14 and 549 per 1,000 with the highest mortality occurred until early 1990^th^. Conversely, mortality in the same age range among individuals without DS remained low and relatively stable at about 2-39 per 1,000. The mortality rate before age 5 among individuals with DS showed a marked decline across birth cohorts, ranging approximately between 14 per 1,000 to 423 per 1,000 live births during the follow up. In contrast, mortality among individuals without DS also declined steadily over time but remained substantially lower throughout, ranging from 2 per 1,000 to 18 per 1,000. For the follow-up period of 6–40 years, among individuals with DS, remained persistently high across the 1967–2018 birth cohorts, ranging approximately between 14 and 549 per 1,000. A higher mortality was observed until the 1990s. Conversely, in the same age range, mortality among individuals without DS remained low and relatively stable, at around 2–39 per 1,000

Figure 2: Trends in mortality rate per 1000 by birth cohort and Down syndrome status among participants with follow up age birth to 40 years, birth to 5 years and 6 to 40 years.

**Factors associated with mortality among populations with Downs syndrome**

The table illustrates factors associated with mortality in individuals with Down syndrome for the follow up restricted to the age of 40 years, birth to 5 years and 6-40 years (Table 2). Our results showed that birth year is the strongest risk factors for mortality in this population. Compared to those born between 2007-2018, individuals born from 1967 to 1976 have the highest hazard ratio (HR: 6.35 [3.90-10.35]), followed by those born from 1977 to 1986 (HR: 3.83 [2.41-6.08]) and from 1987 to 1996 (HR: 2.31 [1.44-3.69]). A birth weight of less than 2,500 grams is associated with increased mortality risk (HR: 1.47 [1.13-1.93]) compared to those with a birth weight of 2,500 grams or more. Individuals born into households with income below the median had a higher mortality risk (HR: 1.41 [1.11-1.79]), compared to those whose parents earned above median income. Having one or more siblings was linked to higher mortality (HR: 1.46 [1.13-1.89]), compared to those with no siblings. Other factors such as sex, gestational age under 38 weeks, maternal age of 30 years or older at pregnancy, unmarried maternal status, and the death of the mother and father during the follow-up period were not statistically significant (Table 2).

In a follow-up restricted to birth to five years, birth year remains the most significant mortality predictor among those with DS. Compared to those born between 2007-2018, the hazard ratios are higher for those born 1967-1976 (HR: 6.18 [3.66-10.43]), born 1977-1986 (HR: 3.61 [2.21-5.91]), and born 1987-1996 (HR: 2.06 [1.25-3.39]). Birth weights under 2,500 grams still present a greater mortality risk (HR: 1.66 [1.24-2.26]) than those with a birth weight of 2,500 grams or more.Having one or more siblings was linked to higher mortality (HR: 1.56 [1.15-2.12]), compared to those with no siblings(Table 2).

For the follow up 6-40 years, individuals born into households with income below the median had a higher mortality risk associated with higher mortality risk (HR: 2.16 [1.20-3.89]) (Table 2) (Table 2).

Table 2: Factors associated with mortality among individuals with Down syndrome for follow up restricted birth to 40 years, birth to 5 years and 6-40 years based on a confounder-adjusted model (Model 1) and a fully adjusted model (Model 2).

Our regression-based models indicated that the increased mortality risk observed among individuals with DS born in the earlier cohorts (1967–1976) for follow-up period from birth to 5 years could be partly explained by covariates such as gestational age, birth weight, household income, maternal age at delivery, maternal marital status, sibling characteristics, and parental death (Table 2). In addition, the observed increase in hazard ratios for the intermediate cohorts (1977–1986 and 1987–1996) compared to 1997-2006 in our fully adjusted model may reflect substantial medical advancements including perinatal and postnatal screening and pediatric care that likely altered health outcomes.

**Discussion**

This study revealed cohort differences in survival among individuals with DS born between 1967 and 2021 with an improved survival among later born cohorts. Across all cohorts, individuals with DS consistently exhibited lower survival probabilities compared to those without the condition, with the survival gap being most pronounced during early childhood. While mortality rates among individuals with DS declined significantly in early life (i.e., before age 5) across birth cohorts, they remained relatively high before age 40. In contrast, individuals without DS experienced better survival and lower mortality rates over the same time span. Low birth weight, having parents with low income, and having one or more siblings were identified as factors that increase mortality among individuals with DS.

The observed improvement in childhood survival rates among more recent birth cohorts with DS is an encouraging trend, reflecting advancements in medical care, early interventions, and improved social support for individuals with the condition. These findings are consistent with trends observed in other high-income countries, including Denmark [21], Sweden [22], the United Kingdom [23], and the United States [24], where access to specialized healthcare and medical innovations has expanded in recent decades. However, survival among individuals with DS remains lower than that of the whole population, indicating that despite overall progress, significant disparities persist. The continued mortality gap highlights the need for sustained efforts to identify and address the health challenges faced by populations living with DS.

Our analyses found a significant association between parental income and DS survival probability, with those born to parents earning below the median income exhibiting a higher risk of mortality. This finding aligns with a growing body of evidence underscoring the profound impact of socioeconomic factors on health outcomes in populations with developmental disabilities [25, 26]. Socioeconomic disadvantages can limit access to quality healthcare services, early interventions, and supportive resources, thereby exacerbating health disparities and mortality risk.

Our findings indicate that individuals with DS born with a low birth weight (<2,500 grams) face a significantly increased risk of mortality than their peers born with normal birth weight. The observed association aligns with previous research demonstrating that low birth weight is a critical determinant of mortality among people with DS [6, 27, 28], often reflecting underlying fetal and neonatal complications and increased vulnerability during infancy and childhood. Furthermore, low birth weight and household income are often interrelated, as socioeconomic deprivation is associated with increased rates of low birth weight [29, 30], which, in turn, contributes to adverse health outcomes. These findings underscore the need for comprehensive approaches addressing both biological and social determinants of health to improve long-term survival among individuals with DS.

The significant decline in survival observed among those with DS during early in life in our study may be attributed to the compounded health challenges faced by infants with DS, including congenital heart defects, respiratory issues, and digestive problems associated with the syndrome [31-33]. Furthermore, secondary analysis indicated that the elevated risk of earlier born individuals partly attenuated after accounting for parental income, maternal age, and birth weight. These findings highlight socioeconomic disadvantages and perinatal vulnerabilities, such as low parental income and low birth weight, play important roles in DS survival disparities.

Our findings indicated that individuals with DS who have one or more siblings demonstrated higher mortality compared to those with no siblings. This may be partly explained by the fact that only children may receive more focused parental attention and resources, such as more joint time, more supervision and help with preventive behaviour, use of healthcare services, financial support, and emotional investment, compared to children with siblings, For individuals with DS, who often require additional medical, educational, and therapeutic support, concentrated parental resources could theoretically enhance access to care, leading to better health management. However, no studies explicitly compare the health outcomes of individuals with DS with and without siblings. Although siblings can positively influence social and emotional development in individuals with DS, it doesn’t necessarily compensate for the parental time spent on helping them with everyday issues, helping them with physical health or attention dilution. Conversely, siblings might compete for parental resources, potentially reducing the time available for specialized care, though this effect is speculative.

While this study provides important insights, some limitations should be noted. First, the data are derived from registry-based sources, which, while comprehensive, may not capture all relevant sociodemographic or health-related factors. Finally, while the study explores the influence of socioeconomic and familial factors in Norway, the familial structure, social support, and healthcare access that influences survival and mortality differ in many countries, particularly low- to middle-income countries which may have a stronger reliance on familial caretaking roles with less healthcare accessibility. In addition, the parents’ life course marital status is not documented. While some studies suggest higher divorce rates among parents of children who have disabilities compared to those whose children without have disabilities [34], other studies have found the opposite [35]. In addition, the results from our flexible parametric model are based on a complete case analysis. Although the proportion of missing data for the variables included in the model was low and sensitivity analyses indicated that the exclusion of incomplete cases did not materially affect the results, the possibility of residual bias due to missing data cannot be entirely ruled out.

In conclusion, there have been significant improvements in survival among individuals with DS from 1967 to 2021. Disparities based on socioeconomic status and perinatal risk factors affect the likelihood of early death among individuals with DS and highlight the need for continued efforts to address these inequalities. The findings underscore the importance of economic support to improve survival with DS. Targeted interventions are required to tackle socioeconomic disparities, and future research should focus on strategies to support individuals with DS and their families in overcoming these challenges.

**Declarations**

**Ethics approval and consent to participate**

This study was approved by the Regional Committee for Medical and Health Research Ethics (Regionale komiteer for medisinsk og helsefaglig forskningsetikk (REK), REK 2019/149 South East). This study was conducted in accordance with the principles of the Declaration of Helsinki.

**Consent for publication**

Not applicable

**Availability of data and materials**

The data used in the current study are available after approval by the Regional Committee for Medical and Health Research Ethics. The data is not publicly available but can be granted upon application to the respective data owners and requires approval from REK.**Competing Interests**

The authors declared no potential conflicts of interest.

**Funding**

This work was supported by the National Institutes of Health (grant number [R01AG069109-01](https://www.sciencedirect.com/science/article/pii/S2666776223001400?via%3Dihub#gs3)) and also an ERC Advanced Grant Project, grant number 101142786 – HOMME. This work was partly supported by the Research Council of Norway through its Centers of Excellence funding scheme (project numbers 2262700). The funding agencies played no role in the design, execution, analysis or interpretation of data, or the writing of this manuscript.

**Authors' contributions**

TM: responsible for the original and final draft of the manuscript, data curation and formal analyses, contributed to the conceptualization, drafting, and reviewing of the final manuscript.

DK: Shared the first authorship with TM and was responsible for the original and final draft of the manuscript, contributed to the conceptualization, drafting, and reviewing of the final manuscript.

BB: contributed to the data curation, conceptualization, drafting, and reviewing of the final manuscript.

HPK: contributed to the conceptualization, drafting, and reviewing of the final manuscript.

GS: contributed to the conceptualization, drafting, and reviewing of the final manuscript.

FKL: contributed to the conceptualization, drafting, and reviewing of the final manuscript.

EML: contributed to the conceptualization, drafting, and reviewing of the final manuscript.

JE: contributed to the conceptualization, drafting, and reviewing of the final manuscript.

ØK: contributed to the conceptualization, drafting, and reviewing of the final manuscript.

AKH: contributed to the conceptualization, drafting, and reviewing of the final manuscript.

BHS: project manager, acquired funding, contributed to the conceptualization, contributed to the conceptualization, drafting, and reviewing of the final manuscript.

contributed to the conceptualization, drafting, and reviewing of the final manuscript.

VS: project administrator, contributed to the conceptualization, contributed to the original draft of the manuscript, acquired funding,

**Acknowledgements**

We are grateful to all participants in the Medical Birth Registry of Norway. This work was funded by the National Institutes of Health (grant number [R01AG069109-01](https://www.sciencedirect.com/science/article/pii/S2666776223001400?via%3Dihub#gs3)), partly supported by the Research Council of Norway ( project numbers 2262700) and an ERC Advanced Grant Project, grant number 101142786 – HOMME.

**References**

1. Akhtar F, Bokhari SRA: **Down syndrome (trisomy 21)**. *StatPearls [*[*https://www*](https://www) *ncbi nlm nih gov/books/NBK526016/]* 2020.

2. Morris JK, Alberman E: **Trends in Down’s syndrome live births and antenatal diagnoses in England and Wales from 1989 to 2008: analysis of data from the National Down Syndrome Cytogenetic Register**. *Bmj* 2009, **339**.

3. Parker SE, Mai CT, Canfield MA, Rickard R, Wang Y, Meyer RE, Anderson P, Mason CA, Collins JS, Kirby RS: **Updated national birth prevalence estimates for selected birth defects in the United States, 2004–2006**. *Birth Defects Research Part A: Clinical and Molecular Teratology* 2010, **88**(12):1008-1016.

4. De Graaf G, Buckley F, Skotko BG: **Estimation of the number of people with Down syndrome in Europe**. *European Journal of Human Genetics* 2021, **29**(3):402-410.

5. Bull MJ: **Down syndrome**. *New England Journal of Medicine* 2020, **382**(24):2344-2352.

6. Glasson EJ, Jacques A, Wong K, Bourke J, Leonard H: **Improved survival in Down syndrome over the last 60 years and the impact of perinatal factors in recent decades**. *The Journal of pediatrics* 2016, **169**:214-220. e211.

7. Huang IC, Brinkman TM, Mullins L, Pui CH, Robison LL, Hudson MM, Krull KR: **Child symptoms, parent behaviors, and family strain in long‐term survivors of childhood acute lymphoblastic leukemia**. *Psycho‐Oncology* 2018, **27**(8):2031-2038.

8. Bruandet A, Richard F, Bombois S, Maurage C, Masse I, Amouyel P, Pasquier F: **Cognitive decline and survival in Alzheimer’s disease according to education level**. *Dementia and geriatric cognitive disorders* 2007, **25**(1):74-80.

9. Livingston G, Huntley J, Liu KY, Costafreda SG, Selbæk G, Alladi S, Ames D, Banerjee S, Burns A, Brayne C: **Dementia prevention, intervention, and care: 2024 report of the Lancet standing Commission**. *The Lancet* 2024, **404**(10452):572-628.

10. McGrath RJ, Stransky ML, Cooley WC, Moeschler JB: **National profile of children with Down syndrome: disease burden, access to care, and family impact**. *The Journal of pediatrics* 2011, **159**(4):535-540. e532.

11. de Graaf G, Levine SP, Goldstein R, Skotko BG: **Parents' perceptions of functional abilities in people with Down syndrome**. *American Journal of Medical Genetics Part A* 2019, **179**(2):161-176.

12. Isgro K: **From a Caretaker's Perspective: Mothers of Children with Down Syndrome as Advocates**. *Women & Language* 2015, **38**(1).

13. Hodapp RM, Urbano RC: **Adult siblings of individuals with Down syndrome versus with autism: findings from a large‐scale US survey**. *Journal of Intellectual Disability Research* 2007, **51**(12):1018-1029.

14. Skotko BG, Levine SP, Goldstein R: **Having a son or daughter with Down syndrome: Perspectives from mothers and fathers**. *American journal of medical genetics part A* 2011, **155**(10):2335-2347.

15. Umberson D, Thomeer MB: **Family matters: Research on family ties and health, 2010 to 2020**. *Journal of Marriage and Family* 2020, **82**(1):404-419.

16. Sherrill BN, Deatrick J, Sanford K: **Family and Health**. *The Wiley Encyclopedia of Health Psychology* 2020:253-262.

17. Winston J, Calhoun LM, Corroon M, Guilkey D, Speizer I: **Impact of the urban reproductive health initiative on family planning uptake at facilities in Kenya, Nigeria, and Senegal**. *BMC women's health* 2018, **18**(1):9.

18. O’Flaherty M, Baxter J, Haynes M, Turrell G: **The family life course and health: Partnership, fertility histories, and later-life physical health trajectories in Australia**. *Demography* 2016, **53**(3):777-804.

19. Cohen PN: **The rising marriage mortality gap among Whites**. 2019.

20. Van den Berg GJ, Gupta S: **The role of marriage in the causal pathway from economic conditions early in life to mortality**. *Journal of health economics* 2015, **40**:141-158.

21. Zhu JL, Hasle H, Correa A, Schendel D, Friedman J, Olsen J, Rasmussen SA: **Survival among people with Down syndrome: a nationwide population-based study in Denmark**. *Genetics in medicine* 2013, **15**(1):64-69.

22. Englund A, Jonsson B, Zander CS, Gustafsson J, Annerén G: **Changes in mortality and causes of death in the Swedish Down syndrome population**. *American Journal of Medical Genetics Part A* 2013, **161**(4):642-649.

23. Irving C, Basu A, Richmond S, Burn J, Wren C: **Twenty-year trends in prevalence and survival of Down syndrome**. *European Journal of Human Genetics* 2008, **16**(11):1336-1340.

24. Dimopoulos K, Kempny A: **Patients with Down syndrome and congenital heart disease: survival is improving, but challenges remain**. In*.*, vol. 102: BMJ Publishing Group Ltd and British Cardiovascular Society; 2016: 1515-1517.

25. Choi B, Yi E: **The Impact of Income and Social Capital on the Health of People with Developmental Disabilities**. In: *Healthcare: 2022*: MDPI; 2022: 1543.

26. Varshney K, Iriowen R, Morrell K, Pillay P, Fossi A, Stephens MM: **Disparities and outcomes of patients living with Down Syndrome undergoing healthcare transitions from pediatric to adult care: A scoping review**. *American Journal of Medical Genetics Part A* 2022, **188**(8):2293-2302.

27. Risnes KR, Vatten LJ, Baker JL, Jameson K, Sovio U, Kajantie E, Osler M, Morley R, Jokela M, Painter RC: **Birthweight and mortality in adulthood: a systematic review and meta-analysis**. *International journal of epidemiology* 2011, **40**(3):647-661.

28. Rankin J, Tennant PW, Bythell M, Pearce MS: **Predictors of survival in children born with Down syndrome: a registry-based study**. *Pediatrics* 2012, **129**(6):e1373-e1381.

29. Mortensen LH: **Socioeconomic inequality in birth weight and gestational age in Denmark 1996–2007: using a family-based approach to explore alternative explanations**. *Social Science & Medicine* 2013, **76**:1-7.

30. Moser K, Li L, Power C: **Social inequalities in low birth weight in England and Wales: trends and implications for future population health**. *Journal of Epidemiology & Community Health* 2003, **57**(9):687-691.

31. Shimokaze T, Toyoshima K, Saito T, Katsumata K, Kemmotsu T, Ishikawa H, Kurosawa K: **Death of children with Down syndrome by gestational age and cause**. *Pediatric Research* 2024, **95**(5):1325-1330.

32. Messick EA, Backes CH, Jackson K, Conroy S, Hart SA, Cua CL: **Morbidity and mortality in neonates with Down Syndrome based on gestational age**. *Journal of Perinatology* 2023, **43**(4):445-451.

33. Seither K, Tabbah S, Tadesse DG, Suhrie KR: **Neonatal complications of Down syndrome and factors necessitating intensive care**. *American journal of medical genetics Part A* 2021, **185**(2):336-343.

34. Lutz W, Kc S: **Global human capital: Integrating education and population**. *Science* 2011, **333**(6042):587-592.

35. Namkung EH, Song J, Greenberg JS, Mailick MR, Floyd FJ: **The relative risk of divorce in parents of children with developmental disabilities: Impacts of lifelong parenting**. *American journal on intellectual and developmental disabilities* 2015, **120**(6):514-526.
